# Early vs Late Coronary Angiography and Intervention Following Thrombolytic Therapy; a Cohort Study

**Published:** 2017-01-11

**Authors:** Bahareh Feizi, Shahram Taghdisi, Jalil Etemadi, Amir Hossein Feizi, Setareh Asgarzadeh, Sepideh Kamal

**Affiliations:** 1 Clinical Research Development Center, Bouali Hospital, Islamic Azad University, Tehran Medical Sciences Branch, Tehran, Iran.; 2 Cardiology Department, Imam Hossein Hospital, Shahid Beheshti University of Medical Sciences, Tehran, Iran.; 3 Clinical Research Development Center, Amir-almomenin Hospital, Islamic Azad University, Tehran Medical Sciences Branch, Tehran, Iran.

**Keywords:** Angiography, thrombolytic therapy, percutaneous coronary intervention, myocardial infarction, postoperative complications

## Abstract

**Introduction:**

The precise time of using percutaneous coronary intervention (PCI) after fibrinolytic therapy for maximum efficiency and minimum side effects is still undetermined. Therefore, the present study was designed to compare the outcome of myocardial infarction (MI) patients who underwent surgical intervention (angiography and PCI) within 48 hours of thrombolytic therapy or after that.

**Methods:**

The present study is a prospective cohort study aiming to compare the occurrence of no-reflow phenomenon, unstable angina, bleeding during intervention, and one month major adverse cardiac outcomes (recurrent MI, need for repeating surgical intervention, and mortality) between MI patents undergoing surgical intervention within the first 48 hours of or after 48 hours of thrombolytic therapy.

**Results:**

90 patients with the mean age of 54.97 ± 10.54 were studied (86.67% male). 50 (56%) patients underwent surgical intervention within 48 hours and 40 (44%) after that. The 2 groups were not significantly different regarding baseline characteristics. No-reflow phenomenon in the < 48 hours group was about twice the > 48 hours group (OR = 0.35; 95% confidence interval: 0.14 – 0.92; p = 0.03), other outcomes were not significantly different. No case of mortality was seen in the 1 month follow up.

**Conclusion:**

Based on the results of the present study, it seems that no-reflow phenomenon rate is significantly lower in patients undergoing surgical intervention after 48 hours of fibrinolytic therapy. The difference between the two groups regarding prevalence of major adverse cardiac outcomes was not statistically significant.

## Introduction

Coronary artery disease (CAD) and its associated side effects are the most common cause of mortality and disability all over the world and its cost burden is the highest among health care costs (1, 2). Currently, each year 900 thousand people are affected with acute myocardial infarction (MI) in the United States, which brings about 225 thousand deaths(3). In the past, open-heart surgery was the major treatment for CAD (4). Yet, in recent years, coronary artery angioplasty through the skin has been proposed as an efficient treatment for ST elevation MI (STEMI)(5). Unfortunately, many STEMI patients either visit hospitals where percutaneous coronary intervention (PCI) is not available, or are not referred to PCI centers in the time range suggested by guidelines (6). In these cases, alternative treatment is using fibrinolytic agents such as tissue plasminogen activator and streptokinase. Although according to the most recent guidelines of American Heart Association (AHA), angiography and PCI should be done within 3 to 24 hours of fibrinolytic therapy, evidence presented in it are derived from level B and C articles, which partially affects its reliability (6). Initial studies have questioned effectiveness of PCI and early angiography after fibrinolytic therapy (7, 8). Yet, the power of the studies carried out in this regard are not high enough to confirm the effectiveness of this treatment (5, -). Consequently, the precise time of using PCI after fibrinolytic therapy for maximum efficiency and minimum side effects is still undetermined. Therefore, the present study was designed to compare the outcome of MI patients who underwent surgical intervention (angiography and PCI) within 48 hours of thrombolytic therapy or after that.

**Table 1:** Baseline characteristics in the studied groups

**Variable**	**First 48 hours (%)**	**After 48 hours (%)**	P
	**Total** (n=50)	**Total **(n=40)	
**Age (year)**	55.5 ± 12.1	54.3 ± 10.9	0.63
**Sex (Male)**	43 (86.0)	35 (87.5)	0.84
**Medical history of**			
Diabetes mellitus	17 (34.0)	14 (35.0)	0.92
Hypertension	22 (44.0)	14 (35.0)	0.39
Hyperlipidemia	20 (40.0)	14 (35.0)	0.63
Cardiovascular accident	7 (14.0)	5 (12.5)	0.84
Myocardial infarction	13 (26.0)	7 (17.5)	0.34
**Smoking**	25 (50.0)	24 (60.0)	0.4

Data are presented as mean ± standard deviation or number and percentage.

**Table 2:** Comparison of patient outcomes in the 2 groups

**Complications**	**First 48 hours (%)**	**After 48 hours (%)**	**P Value**
**Bleeding during intervention**	1 (2)	0 (0.0)	0.56
**No-Reflow phenomenon**	22 (44)	9 (22.5)	0.03
**One month adverse cardiac outcomes**			
Recurrent Myocardial infarction	1 (2)	0 (0.0)	0.56
Repeating revascularization	0 (0.0)	0 (0.0)	---
Mortality	0 (0.0)	0 (0.0)	---

## Methods

***Study design and setting***

The present study is a prospective cohort study aiming to compare the outcome of MI patients who underwent surgical intervention (angiography and PCI) either within the first 48 hours of or after 48 hours of undergoing thrombolytic therapy. The researchers adhered to the principles of Helsinki Declaration throughout the study and the protocol of the study was approved by the Ethical committee of Islamic Azad University, Medical Sciences Branch.

***Participants***

Patients visiting the emergency department (ED) of Bouali Hospital, Tehran, Iran, who were diagnosed with STEMI, were evaluated. Patients over 18 years old, suffering from STEMI, whose symptoms had initiated less than 12 hours ago, and had successful thrombolytic therapy were included. In contrast, those with underlying illnesses such as cancer, chronic hepatic and renal failure (creatinine > 250 mmol/l); pregnant women; drug and alcohol addicts; and those prohibited from using fibrinolytic therapy were excluded. After reaching diagnosis in ED, patients were sent to coronary care unit (CCU), where they underwent thrombolytic therapy and were scheduled for surgical intervention, based on patient condition and preference of in charge cardiologist. Without any interferences from the researches, the participants were followed for 1 month regarding major adverse cardiac outcomes. 1-month follow-up was done by phone calls or in-person. After the follow-up period, patients were divided into 2 groups of < 48 hours and > 48 hours based on time interval between thrombolytic therapy and surgical interventions, and the surgical side effects and one month adverse cardiac outcomes were compared between the 2 groups. The time interval between initiation of fibrinolytic therapy and intervention was 3 to 48 hours in <48 hours group.

***Outcome***

Patients were followed regarding no-reflow phenomenon, unstable angina, and bleeding during intervention, as well as one month major adverse cardiac outcomes including recurrent STEMI or non-ST elevation MI (NSTEMI), need for repeating surgical intervention, and mortality.

***Definitions***

-Recurrent NSTEMI: presence of a Q wave in 2 or more adjacent electrocardiogram leads accompanied by a rise in creatine kinase MB or troponin concentration to above the normal range and more than 50% of the previous measure 18 hours after surgical intervention. 

- Recurrent STEMI: relapse of ischemia symptoms while resting, accompanied by ST elevation of at least 0.1 millivolt in at least 2 adjacent electrocardiogram leads for more than 30 minutes during the first 18 hours after surgical intervention.

- Unstable angina: chest pain while resting, with or without changes in electrocardiogram (ECG) and pain class (based on the classification of Canadian Cardiovascular Society), or presence of dangerous arrhythmias such as atrial tachycardia and atrial fibrillation. 

- No-reflow phenomenon: inadequate muscle perfusion without angiographic evidence of obstruction in a myocardial vessel (12).

Severity of hemorrhage during intervention was determined based on GUST (Global Use of Strategies to Open coronary arteries) severity scale (13). 

***Statistical analysis***

Data were analyzed using SPSS version 20.0. Qualitative data were presented as frequency and percentage and quantitative ones as mean ± standard deviation. Student *t*-test was used for comparing means and chi-square or Fisher’s exact tests for comparing categorical variables. In all analyzes p < 0.05 was considered as significance level.

## Results

90 patients with the mean age of 54.97 ± 10.54 were evaluated (86.67% male). 50 (56%) patients underwent surgical intervention within 48 hours and 40 (44%) after that. Table 1 compares the baseline characteristics of patients in the 2 groups. The 2 groups were not significantly different regarding baseline characteristics. Table 2 compares the frequency of outcomes in the groups, which showed a significant difference only regarding no-reflow phenomenon. This outcome in the < 48 hours group was about twice the > 48 hours group (p = 0.03). The odds ratio for occurrence of no-reflow phenomenon in patients who underwent surgical intervention after 48 hours of fibrinolytic therapy was significantly lower (OR = 0.35; 95% confidence interval: 0.14 – 0.92; p = 0.03).

## Discussion

The findings of this study showed that the frequency of adverse outcomes between MI patients undergoing surgical intervention (angiography and PCI) within the first 48 hours and after 48 hours of receiving thrombolytic agents was not significantly different except for no-reflow phenomenon. In other words, the odds of no-reflow phenomenon was significantly lower in patients who underwent surgical intervention after 48 hours. Although the difference between other studied outcomes was not statistically significant, both cases of major adverse cardiac outcomes were seen in < 48 hours group. No mortality was seen in the 1-month follow-up.

In the years before using stent, early PCI has not been efficient and has been associated with side effects such as hemorrhage, mortality, and coronary artery re-occlusion (14, 15). However, using stents has significantly decreased post-PCI coronary artery re-occlusion incidence (16, 17). This has encouraged doing PCI immediately after fibrinolytic therapy (6, -). As a result, the most recent guideline of AHA has expressed that STEMI patients should undergo PCI as soon as possible. In cases that the patient has undergone fibrinolytic therapy, the ideal time for doing PCI is within the first 24 hours, yet it should not be done in the first 2 – 3 hours (6). In a trial, Armstrong et al. have expressed that early PCI and angiography show better outcomes and fewer side effects compared to late PCI (21). Cantor et al. also believed that early PCI is an efficient therapy with few side effects and the smaller the time interval between fibrinolytic therapy and PCI, the better the results (14). In contrast, the findings of the present study showed that PCI and angiography in the first 48 hours have more side effects compared to the second 48 hours. In line with this study, Collet et al. in their meta-analysis showed that patients who undergo early PCI show more side effects compared to those who undergo intervention after 24 or 48 hours (9). However, a study by Edmond et al. showed that there is no difference between various time intervals of PCI after fibrinolytic therapy, and results and side effects are similar (22). A review article by Capodanno et al. revealed that pharmaco-invasive therapy is effective in STEMI treatment and its time does not make a difference in the outcome of treatment or side effects (23). Vant Hof et al. did not detect a significant difference between early and after 48 hours PCI (24). As can be seen, there is significant difference between study results, which might be due to differences in study design (cohort, clinical trial, or cross-sectional), type of MI, type of thrombolytic used (streptokinase or t-PA), and not paying attention to outcomes such as no-reflow phenomenon. This variation in findings also reveals the need for further studies in this field. 

***Limitations***

The power of the present study was low for comparing mortality rate and other side effects, except for no-reflow phenomenon between the two groups. 1-month follow-up done in the present study only evaluates the short-term adverse outcomes, while long-term follow-ups may enable more accurate assessment. Long-term follow-up and designing further interventional studies is suggested.

## Conclusion:

Based on the results of the present study, it seems that no-reflow phenomenon rate is significantly lower in patients undergoing surgical intervention after 48 hours of fibrinolytic therapy. The difference between the two groups regarding prevalence of major adverse cardiac outcomes was not statistically significant. 
